# The chromosome-level Stevia genome provides insights into steviol glycoside biosynthesis

**DOI:** 10.1038/s41438-021-00565-4

**Published:** 2021-06-01

**Authors:** Xiaoyang Xu, Haiyan Yuan, Xiaqing Yu, Suzhen Huang, Yuming Sun, Ting Zhang, Qingquan Liu, Haiying Tong, Yongxia Zhang, Yinjie Wang, Chunxiao Liu, Lei Wu, Menglan Hou, Yongheng Yang

**Affiliations:** 1 Institute of Botany, Jiangsu Province and Chinese Academy of Sciences/Jiangsu Provincial Platform for Conservation and Utilization of Agricultural Germplasm, Nanjing, 210014 Jiangsu China; 2 College of Horticulture, Nanjing Agricultural University, Nanjing, 210095 Jiangsu China; 3 Institute of Pomology, Jiangsu Academy of Agricultural Sciences/Jiangsu Key Laboratory for Horticultural Crop Genetic Improvement, Nanjing, 210014 Jiangsu China; 4 Biomarker Technologies Corporation, Beijing, 101300 China

**Keywords:** Genome, Plant sciences

## Abstract

Stevia (*Stevia rebaudiana* Bertoni) is well known for its very sweet steviol glycosides (SGs) consisting of a common tetracyclic diterpenoid steviol backbone and a variable glycone. Steviol glycosides are 150–300 times sweeter than sucrose and are used as natural zero-calorie sweeteners. However, the most promising compounds are biosynthesized in small amounts. Based on Illumina, PacBio, and Hi-C sequencing, we constructed a chromosome-level assembly of Stevia covering 1416 Mb with a contig N50 value of 616.85 kb and a scaffold N50 value of 106.55 Mb. More than four-fifths of the Stevia genome consisted of repetitive elements. We annotated 44,143 high-confidence protein-coding genes in the high-quality genome. Genome evolution analysis suggested that Stevia and sunflower diverged ~29.4 million years ago (Mya), shortly after the whole-genome duplication (WGD) event (WGD-2, ~32.1 Mya) that occurred in their common ancestor. Comparative genomic analysis revealed that the expanded genes in Stevia were mainly enriched for biosynthesis of specialized metabolites, especially biosynthesis of terpenoid backbones, and for further oxidation and glycosylation of these compounds. We further identified all candidate genes involved in SG biosynthesis. Collectively, our current findings on the Stevia reference genome will be very helpful for dissecting the evolutionary history of Stevia and for discovering novel genes contributing to SG biosynthesis and other important agronomic traits in future breeding programs.

## Introduction

High-sugar diets are known to cause severe health problems such as obesity and diabetes^1^. Some countries have levied sugar taxes to reduce the consumption of high-calorie sugars, a recommended strategy to reduce sugar consumption by encouraging substitution with zero-calorie sweeteners^2^. Steviol glycosides, extracted from the leaves of Stevia, contain no calories and have desirable natural sweetness^3^. *Stevia rebaudiana* (2*n* = 22) is a sweet herb native to Paraguay, and its leaf extract has been used as a natural sweetener for centuries in South America^4^. In addition to sweetness, the two abundant components of SGs, stevioside and rebaudioside A (Reb A), may also provide therapeutic benefits for type 2 diabetes, as these compounds can directly enhance insulin secretion by potentiating TRPM5 channel activity in animal models^5,6^. Stevia is widely cultivated in Asia, North America, and Europe for its use as a natural sweetener and traditional medicine.

The genus *Stevia* belongs to the Eupatorieae tribe within the Asteraceae family. Among the ~230 species of the genus *Stevia*, *S. rebaudiana* is the only one that contains SGs^3,7^. SGs have a core diterpenoid steviol backbone (aglycone) decorated with different glycosylation patterns at the C-13 and C-19 positions^3,8^. These diterpenoid glycosides occur almost exclusively in Stevia leaves, accounting for up to ~20% of the dry weight^9,10^. Stevioside and Reb A are the two main components of SGs, followed by Reb C, Reb F, dulcoside A, Reb D, and Reb M. Labeling experiments have revealed that the backbone of SGs is biosynthesized from 5-carbon isoprenoid units, which are predominantly derived from the methylerythritol phosphate (MEP) pathway^11^. The biosynthetic pathways of SG and gibberellic acid (GA) share four steps, and the last common substrate of these two labdane-type diterpenoids is *ent*-kaurenoic acid^12–14^. In the SG biosynthesis pathway, *ent*-kaurenoic acid hydroxylase (*ent*-KAH) catalyzes the 13-hydroxylation of *ent*-kaurenoic acid to form *ent*-13-hydroxy kaurenoic acid (steviol), which serves as the backbone for all SGs^14,15^. Then, a series of glycosylation processes of the aglycone (steviol) catalyzed by a set of cytosolic UDP-dependent glycosyltransferases (UGTs) leads to production of diverse types of SGs. Glucose is a major sugar moiety in all SGs, while rhamnose and xylose are present in only a few SGs, such as Reb C and dulcoside A^3^.

Stevia is unique in its accumulation of SGs, which are secondary metabolites of diterpenoids and have initial biosynthetic steps similar to those of GAs. GAs are essential for normal plant growth and development, and genes involved in the GA biosynthesis pathway are conserved and strictly regulated in higher plants^16,17^. It seems that the biosynthesis of SGs and GAs should be spatially or temporally separated to avoid disturbing the normal metabolism of GAs^12^; however, the evolution of SG accumulation and the separation mechanisms of SG and GA biosynthesis in Stevia remain elusive. After the formation of the core tetracyclic diterpenes (GA_12_ and steviol), oxidation and glycosylation take place to yield the final bioactive compounds: GAs and SGs, respectively. Unlike the oxidase genes involved in GA biosynthesis, UGT genes participating in diterpenoid glycosylation have rarely been documented^18,19^. Stevia is an ideal plant model for the study of diterpenoid glycosylation, not only because its leaves accumulate more than 30 types of SGs but also because of its short growth cycle and easy reproduction. Due to the absence of the genome sequence of Stevia, most studies identifying UGT genes involved in glycosylation of SGs have been based on expressed sequence tags or transcriptomic sequences, and only three UGTs have been characterized to contribute to the biosynthesis of SGs so far^20,21^. This deficiency has hindered research on SG biosynthesis and, consequently, has hindered comprehensive understanding of the evolution of Stevia in Asteraceae.

In the present study, we generated a high-quality reference genome sequence for Stevia (cv. ‘Zhongshan No. 7’) through a combination of PacBio sequencing and Hi-C approaches. Based on this genome sequence, we performed an evolutionary analysis of Stevia in the Asteraceae family. Furthermore, candidate gene sets involved in SG biosynthesis were identified. This reference genome will be very helpful for the evolutionary understanding of SG biosynthesis and for quality improvement of Stevia in the future.

## Results

### Genome sequencing and assembly

The leaves of the Chinese Stevia cultivar ‘Zhongshan No. 7’ were collected for de novo genome sequencing and assembly. Based on K-mer analysis, we estimated a genome size of 1.16 Gb for Stevia, with heterozygosity rates of 0.43% and 73.13% repeats (Supplementary Table 1 and Supplementary Fig. 1). To accurately assemble this complex genome with a high rate of repeat sequences, we used a combination of short-read Illumina sequencing, long-read PacBio sequencing, and Hi-C sequence approaches. We obtained 114.96 Gb of PacBio sequencing subreads, providing ~99.5-fold coverage of the Stevia genome (Supplementary Table 2). These subreads were assembled into a 1405 Mb genome that contained 6978 contigs, with a contig N50 value of 616.85 kb, and the longest contig was 26.27 Mb in length (Supplementary Table 3). A total of 76.86 Gb of Hi-C clean data were generated, of which 90.42% reads were mapped to the assembled contigs (Supplementary Table 4). Under the guidance of the Hi-C data, we successfully clustered 6358 contigs into 11 pseudochromosomes and oriented 91.28% of the assembly according to a hierarchical clustering strategy^22^ (Supplementary Table 5 and Supplementary Fig. 2). The final chromosome-level Stevia assembly was 1416 Mb in length, with an N50 scaffold size of 106.55 Mb (Table 1).

**Table 1:** Statistics of the final Stevia genome

	Stevia (PacBio + Hi-C)
Assembly feature	
Assembly length	1416 Mb
Number of scaffolds	3708
Scaffold N50	106.55 Mb
Number of contigs	6735
Contig N50	616.85 Kb
Longest contig	22.53 Mb
GC content	36.98%
Gap %	0.013%
Genome annotation	
Repetitive sequences	1134 Mb (80.11%)
Protein-coding genes	44,143
Average gene length	3493 bp

We used a combination of three data sources to assess the completeness of the Stevia genome assembly. First, we aligned our Illumina data to the assembled genome, and 98.14% of the clean reads were mapped (Supplementary Table 6). A total of 451 of the 458 core eukaryotic genes (CEGs) were identified in our current Stevia genome assembly (Supplementary Table 7). Moreover, BUSCO^23^ analysis revealed that 86.04% of the complete BUSCOs were present in the Stevia assembly (Supplementary Table 8). All these findings suggested that the assembled Stevia genome had high completeness and accuracy.

### Annotation of the Stevia assembly

**Fig. 1:**
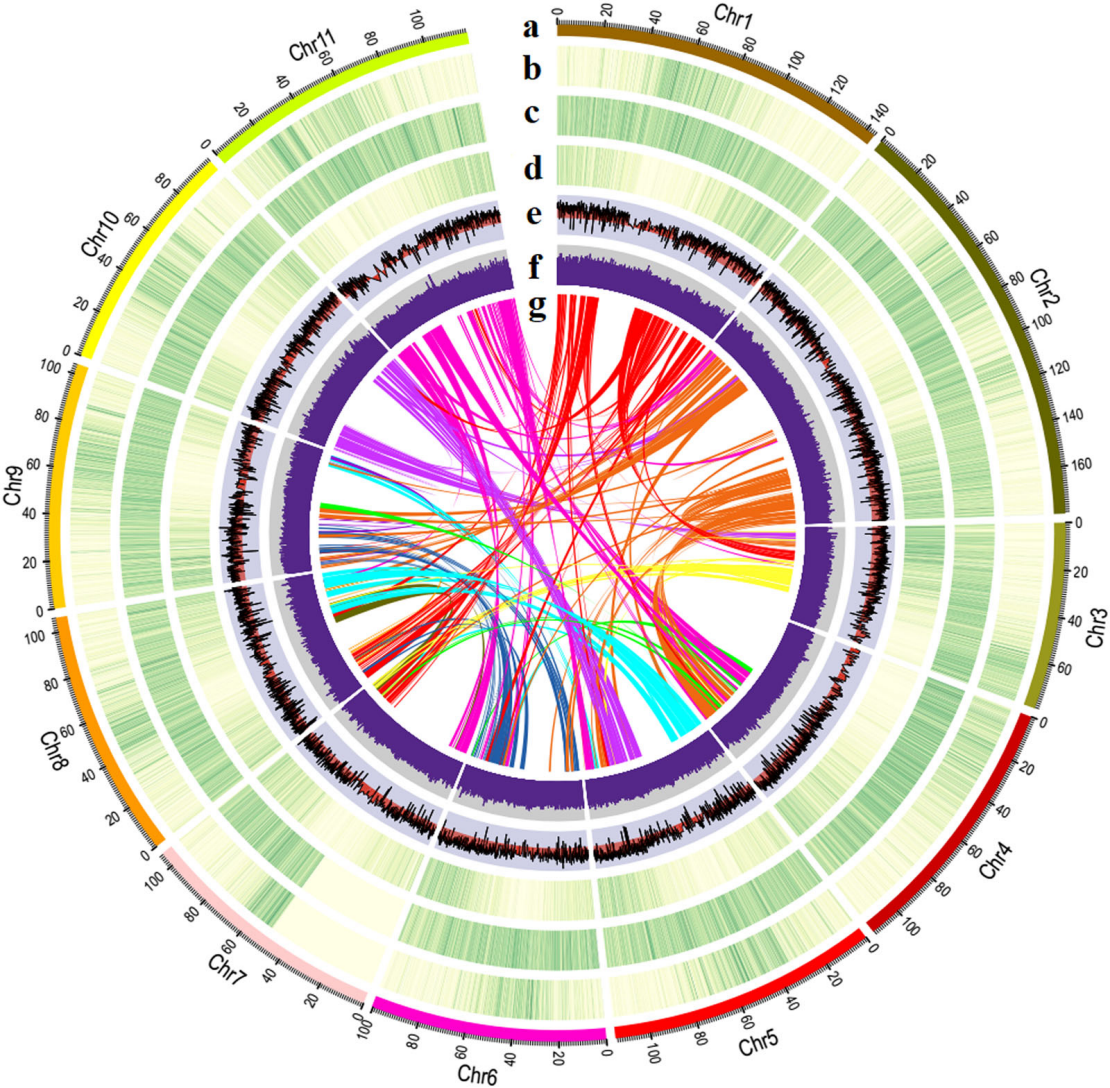
Characterization of the Stevia genome. **a** Circular representation of the pseudomolecules (Mb). **b** Density of *Copia* LTR-RTs. **c** Density of *Gypsy* LTR-RTs. **d** Gene density. **e** Average transcript levels, log_2_(RPKM). **f** GC content. **g** Syntenic blocks across Stevia pseudomolecules. RPKM reads per kb per million reads

We annotated the repetitive elements of the Stevia genome through homology-based methods and in silico prediction, and 80.11% of the assembly was determined to be composed of repetitive elements. Among them, retrotransposons accounted for 69.45%, and DNA transposons accounted for 5.83%. More than 65% of the Stevia genome consisted of long terminal repeat retrotransposons (LTR-RTs), 32.30% of which belonged to the *Copia* lineage and 66.76% of which belonged to the *Gypsy* lineage (Supplementary Table 9). It was not surprising that such a high content of LTR-RTs was present in the Stevia genome since LTR-RTs are also present in large proportions in other Asteraceae species, such as *Helianthus annuus* (sunflower)^24^, *Lactuca sativa* (lettuce)^25^, and *Chrysanthemum nankingense*^26^. For protein-coding gene annotation, we used a combination of three methods: homology-based, ab initio, and RNA Seq-assisted prediction methods. Finally, 44,143 protein-coding genes were predicted in our current Stevia assembly, with an average gene length of 3493 bp (Table 1). Transcriptome analysis showed that 37,489 predicted genes (84.93%) were supported by at least one of the seven organs (root, stem, and leaves at five different developmental stages). Overall, 41,801 protein-coding genes (94.69%) were assigned functions in at least one of the five databases (NR, TrEMBL, KOG, KEGG, and GO) (Supplementary Table 10), and 40,355 were anchored on the 11 pseudochromosomes. Fig. 1b–e shows the *Copia* density, *Gypsy* density, gene density, and transcriptional level of each pseudochromosome.

### Evolutionary history of *S. rebaudiana*

**Fig. 2:**
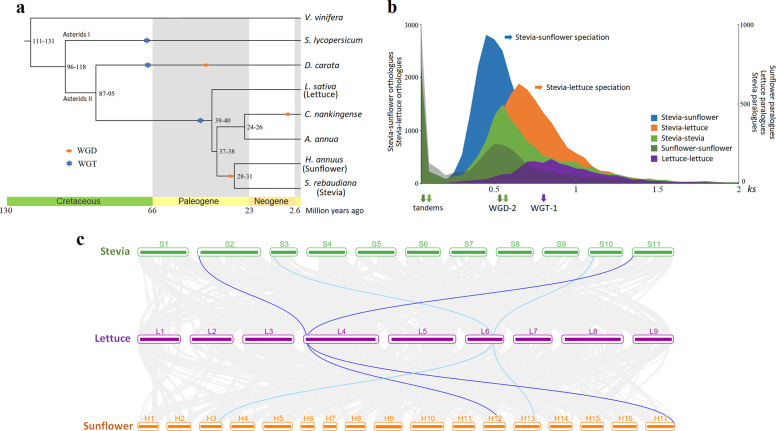
Species phylogenetic tree and genome evolution of Stevia. **a** Phylogenetic tree of Stevia and seven other plants based on 799 single-copy orthologous genes. **b** *Ks* distributions. Left *y*-axis, Stevia-sunflower orthologues (blue), Stevia-lettuce orthologues (orange); right *y*-axis, Stevia paralogues (green), sunflower paralogues (dark green), lettuce paralogues (purple). **c** Synteny blocks of Stevia-lettuce-sunflower

To understand the evolutionary relationship between Stevia and other Asteraceae plants, we performed a comparative genomic analysis using *Vitis vinifera*, *Solanum lycopersicum*, *Daucus carota*, and five Asteraceae plants (*L. sativa*, *C. nankingense*, *Artemisia annua*, *H. annuus*, and *S. rebaudiana*). Phylogenetic analysis based on 799 single-copy orthologous genes identified in these eight species confirmed the close relationship between Stevia and sunflower (Heliantheae alliance) and between *C. nankingense* and *A. annua* (Anthemideae tribe) (Fig. 2a). The estimated divergence time of Stevia and sunflower was ~28–31 million years ago (Mya). The most recent common ancestor (MRCA) of Stevia and sunflower diverged from the MRCA of *C. nankingense* and *A. annua* ~37–38 Mya, which is consistent with the findings of Song et al.^26^. The MRCA of Stevia and *C. nankingense* diverged from lettuce ~39–40 Mya (Fig. 2a).

We further investigated whole-genome duplication (WGD) events during Stevia evolution since WGD has been considered a significant source of plant genetic, biochemical, and evolutionary novelty^27–29^. We identified 5209 paralogous gene pairs that accounted for 20.69% of the predicted Stevia genes using the MCScanX package^30^. The *Ks* distribution of these duplicated gene pairs peaked at 0.53, reflecting the occurrence of a WGD event ~32.1 Mya (Fig. 2b). Based on the *Ks* distribution of the orthologous gene pairs between Stevia and sunflower, we estimated that their divergence time was ~29.4 Mya, indicating that they diverged soon after the WGD event (WGD-2) experienced by their common ancestor^24^. The *Ks* distribution of homologous gene pairs clearly illustrated that whole-genome triplication (WGT-1, ~45.5–51.5 Mya) occurred in lettuce (Fig. 2b), which is also believed to have occurred in the ancestry of most Asteraceae^24–26,31^. This analysis showed that Stevia experienced a complicated evolutionary history characterized by a recent WGD-2 shared with sunflower, the basal WGT-1 in Asteraceae, and the ancestral paleohexaploidy event (WGT-γ) that occurred in all eudicots^32^. Thus, for any ancestral region from the MRCA of Asteraceae (post-WGT-1), two inherited regions are currently expected to exist in the Stevia and sunflower genomes compared to the lettuce genome (Fig. 2c). Although Stevia and sunflower experienced the same paleopolyploidy events (WGT-γ, WGT-1, and WGD-2) and there were many collinear regions between their genomes (Supplementary Fig. 3), these two species may have undergone different chromosome rearrangement patterns and duplicated gene loss after divergence, resulting in different chromosome numbers and genome sizes.

### Gene family analysis

**Fig. 3:**
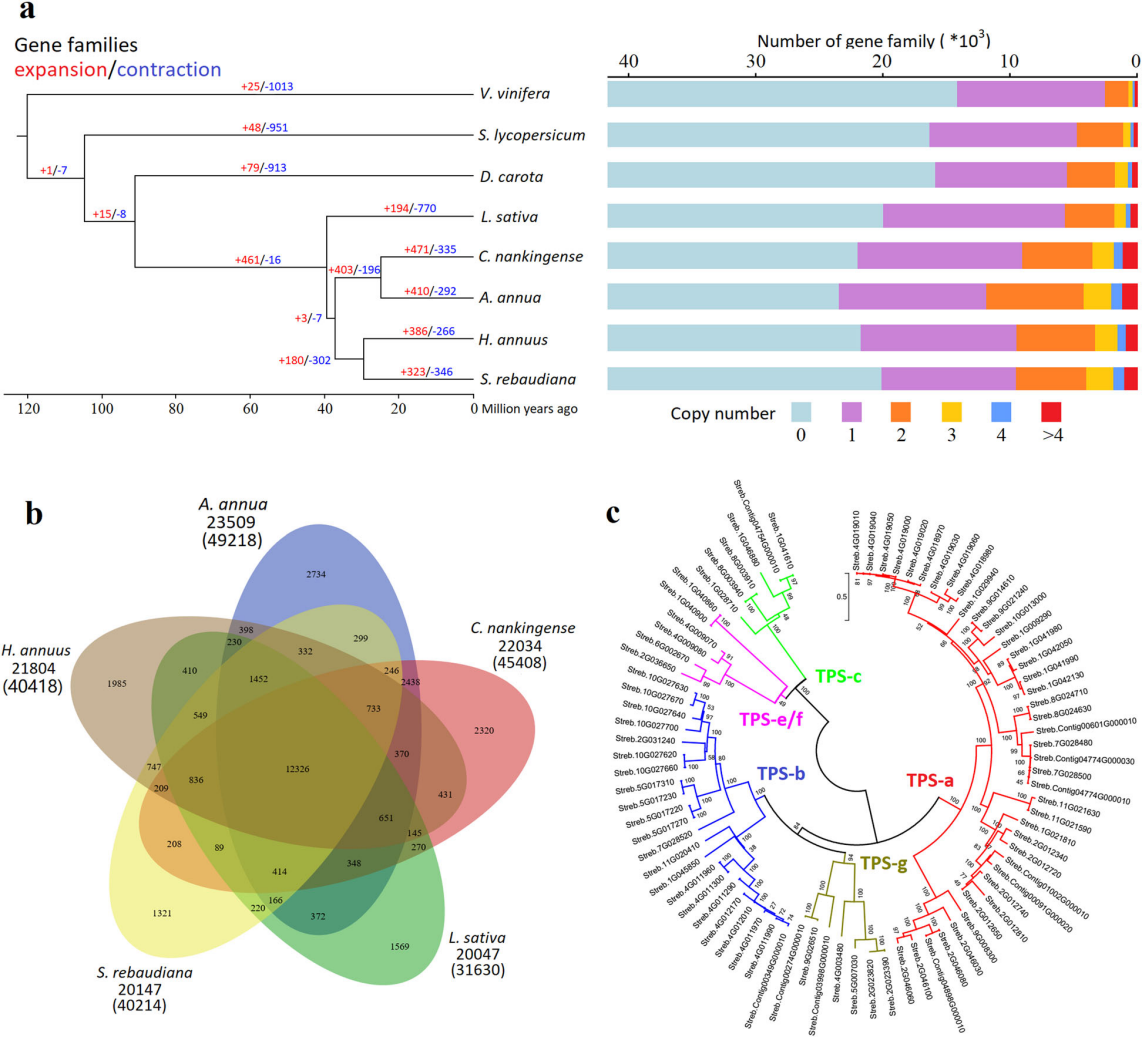
Gene families analyses in the Stevia genome. **a** The red and blue numbers mapped to the species phylogenetic tree indicate gene families that underwent expansion and contraction, respectively. **b** Venn diagram of shared gene families in Stevia and four other plants of Asteraceae. **c** Phylogenetic tree of the terpene synthase (TPS) gene family in Stevia. The TPS subfamilies are labeled

Based on sequence homology, a total of 41,701 gene families containing 276,277 genes were identified using the predicted genes of the above eight plants (seven from asterids and *V. vinifera* as an outgroup) (Fig. 3a and Supplementary Table 11). Of these, 5749 gene families consisting of 68,964 genes were shared among all eight plants, and 12,326 were shared among the five Asteraceae plants (Fig. 3b). We assigned 40,214 Stevia genes to 20,147 families and found that 1057 gene families contained 4281 genes unique to Stevia. Gene Ontology (GO) enrichment analysis revealed that these unique gene families were mostly involved in RNA-directed DNA polymerase activity (GO:0003964), aspartic-type endopeptidase activity (GO:0004190), and RNA binding (GO:0003723) (Supplementary Fig. 4).

Further gene family analysis demonstrated that 323 gene families were expanded in the Stevia genome, while 346 were contracted (Fig. 3a). GO enrichment analysis of the expanded gene families of Stevia revealed that they were enriched for transferase activity (GO:0016758), monooxygenase activity (GO:0004497), ADP binding (GO:0043531), catalytic activity (GO:0003824), and terpene synthase activity (GO:0010333) (Supplementary Fig. 5). KEGG enrichment analysis revealed that the expanded gene families of Stevia were enriched mainly for phenylpropanoid biosynthesis (ko00940), terpenoid backbone biosynthesis (ko00900), flavonoid biosynthesis (ko00941), monoterpenoid biosynthesis (ko00902), cyanoamino acid metabolism (ko00460), and sesquiterpenoid and triterpenoid biosynthesis (ko00909) (Supplementary Fig. 6). The GO and KEGG enrichment analyses demonstrated that a considerable number of these expanded gene families participated in the biosynthesis of specialized metabolites. As the terpenoid biosynthesis pathway was enriched several times, we further investigated the expansion pattern of the terpene synthase (TPS) gene family, which drives the diversification of terpenoids. Eighty-two TPS genes were identified in the Stevia genome, which could be divided into five subfamilies. More than three-quarters of the Stevia TPS genes were classified into the TPS-a and TPS-b subfamilies, indicating significant expansion of these two subfamilies (Fig. 3c).

### Genes involved in SG biosynthesis

**Fig. 4:**
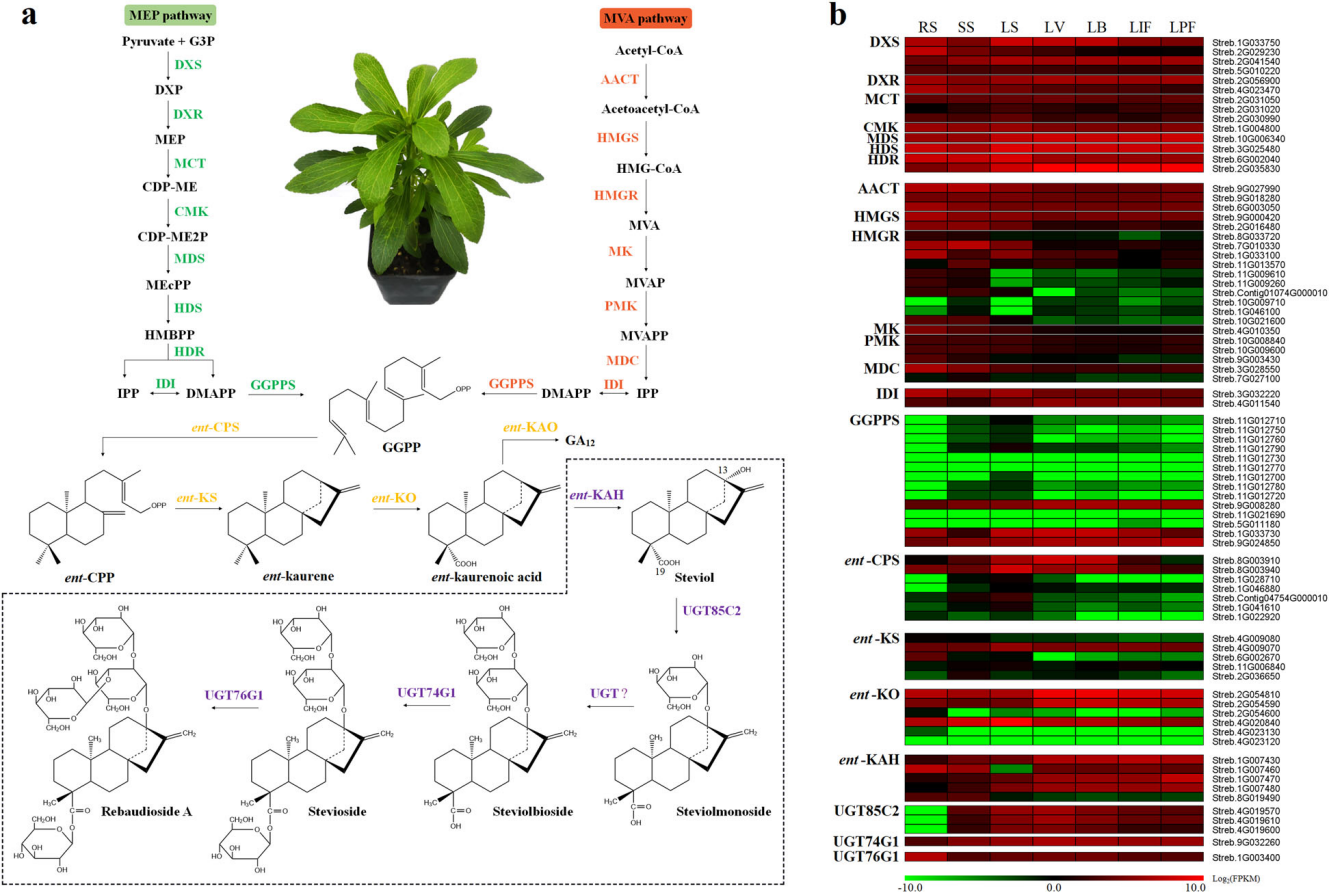
The SGs biosynthesis pathway in Stevia. **a** Diagram depicting the pathway of SGs biosynthesis. Inside the dashed box is the unique SGs biosynthesis pathway in Stevia. **b** Expression patterns of candidate genes of the SGs biosynthesis pathway. RS root at the seedling stage, SS stem at the seedling stage, LS leaf at the seedling stage, LV leaf at the vegetative stage, LB leaf at the bud stage, LIF leaf at the initial flowering stage, LPF leaf at the peak flowering stage

Accumulation of large amounts of SGs in leaves is the most notable feature of Stevia. Although the SG biosynthetic pathway has been extensively studied during the past two decades, and although some of the critical UGT genes have been well characterized^21,33^, we obtained new insights into SG biosynthesis by combining genomic and transcriptome analyses. All terpenes are derived from 5-carbon isoprenoid units produced through either the MEP pathway or the mevalonate (MVA) pathway^34^. Candidate genes in the MEP and MVA pathways were identified using homolog searching and functional annotation methods. Transcriptome analysis revealed that almost all the candidate genes in the MEP pathway were expressed in seven selected tissues, including leaves at different developmental stages, with high accumulation of SGs (Fig. 4). However, the expression levels of *HMGR* and *MK* in the MVA pathway were deficient in the leaves, indicating that the 5-carbon isoprenoid unit for SG biosynthesis comes from mainly the MEP pathway instead of the MVA pathway, which is consistent with the conclusions derived from the results of labeling experiments^11^.

Since the biosynthesis of SGs shares four steps with the biosynthesis of GAs before the generation of *ent*-kaurenoic acid, we identified all candidate genes involved in this common pathway, including 14 geranylgeranyl diphosphate (GGPP) synthase (GGPPS) genes, seven *en*t-copalyl diphosphate synthase (*ent*-CPS) genes, five *ent*-kaurene synthase (*ent*-KS) genes, and six *ent*-kaurene oxidase (*ent*-KO) genes (Fig. 4). All four types of genes were multicopy genes in the Stevia genome, and the homologous genes showed expression differentiation (Fig. 4b), reflecting subfunctionalization or neofunctionalization of these duplicated genes. Stevia has evolved to biosynthesize SGs based on the conserved early steps of the GA biosynthesis pathway in vascular plants.

Steviol glycoside biosynthesis diverges from GA biosynthesis with the 13-hydroxylation of *ent*-kaurenoic acid by *ent*-KAH. The evolution of *ent*-KAH from numerous P450s was the key step of SG biosynthesis in Stevia; however, cloning of its encoding gene has not been successful yet^3,35^. Several potential *ent*-KAH genes of Stevia have been deposited in the NCBI database, and most belong to the CYP716 family. In contrast, CYP714A2, a member of the CYP714 family of *Arabidopsis thaliana*, has been reported to catalyze the 13-hydroxylation of *ent*-kaurenoic acid when expressed in yeast^36^. Thus, we identified all putative members of the CYP716 and CYP714 families in the Stevia genome. There was significant expansion of CYP716 genes, and four tandemly duplicated genes (*Streb.1G007430*, *Streb.1G007460*, *Streb.1G007470*, and *Streb.1G007480*) were consistently highly expressed in leaves with SG biosynthesis (Supplementary Fig. 7). In contrast, there was only one member of the CYP714 family in the Stevia genome (*Streb.8G019490*), and it was hardly expressed in the leaves (Fig. 4b). After the formation of steviol, continuous glycosylation processes catalyzed by a set of UGTs lead to different types of SGs (Fig. 4a). UGT genes were highly enriched (GO:0016758, *P* < 0.001) among the expanded gene families of Stevia. In total, we identified 259 putative UGT genes in the Stevia genome, and 86 UGT genes were expressed in at least two of the five selected leaf tissues (Supplementary Fig. 8), including three UGT genes that have been reported to be involved in SG biosynthesis (*UGT85C2*, *UGT74G1*, and *UGT76G1*). Unearthing of these candidate UGT genes through genomic and transcriptome analyses will accelerate the identification of UGT genes involved in specific SG glycosylation.

## Discussion

Obesity is a serious global health issue that affects millions of people, with a high-sugar diet being one of the leading causes of obesity. Reducing sugar intake by substitution with zero-calorie sweeteners is an effective way to reduce dietary energy consumption. Although artificial sweeteners such as saccharin, aspartame, and sucralose are widely added to various food products in daily use, long-term uptake of these sugar substitutes may pose health risks^37,38^. There is a strong demand for natural zero-calorie sweeteners, and SGs may be the most promising candidates. Steviol glycosides have been approved by the foremost regulatory authorities worldwide for use in foods and beverages. High yields and improvements in the levels of the best-tasting SGs, such as Reb A, Reb D, and Reb M, are currently the main objectives for breeding of Stevia.

Thus far, there have been no comprehensive analyses combining genomic and transcriptomic methods to provide in-depth insights into the unique diterpenes (SGs) of Stevia. It is still very challenging to construct a high-quality Stevia genome assembly based only on second-generation sequencing owing to the large size and high complexity of the genome as well as the percentage of repeats^39^. Here, we propose a chromosome-level genome assembly of Stevia. The Stevia genome spanning 1416 Mb was obtained, with a contig N50 of 616.85 kb and a scaffold N50 of 106.55 Mb. We predicted 44,143 protein-coding genes in our current assembly using homology-based, ab initio, and RNA Seq-assisted prediction methods; this number is almost twice that of the previous genome assembled using second-generation short sequences (24,994), probably because only 411 Mb of the genome had previously been assembled^39^. Therefore, the genome assembled using long sequences and Hi-C approaches in this study is superior to the genome previously assembled using only short sequences.

More than four-fifths of the Stevia genome consisted of repetitive elements, of which 21.02% belonged to the *Copia* lineage and 43.44% belonged to the *Gypsy* lineage. In sunflower, more than three-quarters of the genome was composed of LTR-RTs, 59.9% of which belonged to the *Gypsy* lineage and 25.8% of which belonged to the *Copia* lineage^24^. In *C. nankingense*, repetitive elements accounted for 69.6% of the genome, among which LTR-RTs (*Gypsy* and *Copia*) were the most abundant^26^. Having many repetitive sequences, especially LTR-RTs, might be a significant feature of the Asteraceae family, contributing to the genome sizes of its members.

Comparative genomic analyses of Stevia and other Asteraceae plants have provided crucial clues regarding Stevia genome evolution in Asteraceae. Our results showed that Stevia and sunflower diverged ~29.4 Mya, shortly after the WGD event (WGD-2, ~32.1 Mya) that occurred in their MRCA (Fig. 2a, b). Stevia, a member of the Asteraceae family, also experienced a basal WGT-1 event in Asteraceae, and a WGT-γ event occurred in all eudicots^24,25,32^. Most of the syntenic blocks present in the Stevia genome were derived from the recent WGD-2 event, while syntenic blocks derived from ancient WGT-1 events were rarely preserved (Fig. 2b). After it diverged from an ancestor shared with sunflower, Stevia evolved to synthesize SGs, unique metabolites not found in other plants. The expansion of specific gene families may play important roles in promoting phenotypic diversification as well as in the evolution of novel traits in plants^40,41^. The expanded genes in Stevia were mainly enriched for biosynthesis of specialized metabolites, especially biosynthesis of terpenoid backbones, and for further oxidation and glycosylation of these compounds (Supplementary Figs. 5, 6). We further identified all candidate genes in the pathway of SG biosynthesis based on the genome sequences and found that the essential genes responsible for steviol biosynthesis were multiple-copy genes (Fig. 4b). These duplicated genes might be important contributors to the ability of Stevia to synthesize SGs. Thus, this high-quality chromosome-level genome assembly will undoubtedly benefit researchers in the exploration of Stevia characteristics.

## Materials and methods

### Leaf collection, DNA library construction, and genome sequencing

Fresh young leaves present during the seedling stage of ‘Zhongshan No. 7’, a cultivated diploid Stevia species, were collected from the Stevia germplasm resource laboratory located at the Nanjing Botanical Garden Mem. Sun Yat-Sen. Genomic DNA was isolated for Illumina and PacBio sequencing. For Illumina sequencing, a short-read (270 bp) library was constructed and sequenced on an Illumina HiSeq platform (Illumina, CA, USA), and 141.90 Gb of clean reads were obtained. For PacBio sequencing, genomic DNA was fragmented to ~20 kb to construct a long-read library according to the manufacturer’s instructions (Pacific Biosciences, CA, USA), and then the library was sequenced on a PacBio Sequel platform. After filtering out the low-quality reads and sequence adapters, we obtained 114.95 Gb of clean subreads with an N50 value of 12.82 kb.

### Genome assembly

For de novo genome assembly, we first used Canu (v1.5)^42^ to correct for potential errors in the PacBio subreads. Then, the high-quality PacBio subreads were independently assembled using WTDBG (v1.2.8), FALCON (v0.7)^43^ and Canu (v1.5). These three assembly strategies yielded 1.36, 3.25, and 2.03 Gb assemblies, and the contig N50 sizes of these three assemblies were 205.51, 59.71, and 277.70 kb, respectively. We further merged the well-assembled WTGDB and Canu assemblies using Quickmerge^44^. To correct the indel and SNP errors in the assembly sequence, we mapped paired-end Illumina reads to the merged assembly using Pilon^45^. Finally, the size of the genome assembled using PacBio long reads was 1.40 Gb with a contig N50 value of 616.85 kb (Supplementary Table 3). To evaluate the quality of the assembly, we used BWA^46^ to map the short paired-end reads to the optimized contigs and then performed CEGMA^47^ and BUSCO^23^ analyses.

### Chromosome-level assembly

Hi-C sequencing for the chromosome-level genome assembly was performed as previously described^48^. Briefly, fresh young leaves present at the seedling stage of ‘Zhongshan No. 7’ were collected and fixed in formaldehyde solution. *Hin*dIII was used to digest the chromatin extracted from the fixed leaves. The DNA fragments were then ligated together to form chimeric junctions after biotinylation. Next, the enriched chimeric junctions were physically sheared into DNA fragments of 300–700 bp in length. Biotin-containing DNA fragments were enriched through streptavidin pulldown and then subjected to Illumina HiSeq sequencing. Finally, ~76.86 Gb of clean Hi-C reads were generated.

HiC-Pro^49^ was used to assess the Hi-C sequencing data. The Hi-C sequencing data were mapped to assembled contigs using BWA-aln^46^. The preassembled scaffolds were split into 50 kb segments on average and conjoined with unique mapped reads for assembly using LACHESIS software^22^. To evaluate the final chromosome assemblies, we divided them into bins of equal lengths (100 kb) and visualized the interaction matrix in a heat map.

### Genome annotation

De novo searches and homology-based alignments were used to predict repetitive sequences across the Stevia genome. We first used PILER-DF (v2.4)^50^, RepeatScout (v1.0.5)^51^, and LTR_FINDER (v1.05)^52^ to predict de novo repetitive sequences and classified them into families using PASTEClassifier^53^. We then used RepeatMasker (v4.0.6)^54^ to scan the integrated database of the de novo repetitive sequences and the known Repbase^55^ TE library.

We used homology-based, ab initio, and RNA Seq-assisted approaches to predict protein-coding genes in the Stevia genome assembly. Augustus^56^, GlimmerHMM^57^, SNAP^58^ and GeneID^59^ were used for ab initio programs. In homologous predictions, the protein sequences of *A. thaliana*, *H. annuus*, *L. sativa*, and *Oryza sativa* from Phytozome were downloaded and aligned to the assembled Stevia genome using TBLASTN^60^. We then aligned the homologous genomic sequences against matching proteins to construct the exact protein-coding gene models using GeMoMa^61^. For the RNA Seq-assisted predictions, RNA sequencing reads from different organs of Stevia were mapped to the assembly, and transcripts from these mapping results were identified using GeneMarkS-T^62^ and TransDecoder (https://github.com). We integrated all gene models obtained from the above three annotation procedures with EVM^63^ to construct a final consensus set and then filtered it with PASA (v2.0.2)^64^. These predicted protein-coding genes were then assigned to BLAST against public databases, including TrEMBL^65^ and NCBI nonredundant protein databases. Blast2GO^66^ was used to determine the functions and pathways based on the GO^67^ and KEGG^68^ databases.

### Genome evolution and gene family analysis

Orthologous groups among the eight plants (*V. vinifera*, *S. lycopersicum*, *D. carota*, *L. sativa*, *C. nankingense*, *A. annua*, *H. annuus*, and *S. rebaudiana*) were identified using OrthoMCL^69^. All-versus-all comparisons were performed using BLASTP (*E*-value: 1e−05), and orthologous groups were clustered using OrthoMCL. We used MAFFT^70^ to align the protein sequences of 799 single-copy genes, removed the poorly aligned regions with Gblocks^71^, and concatenated the alignment results for phylogenetic analysis using RAxML (v8.0.0)^72^. We estimated the species divergence times using MCMCTREE (v4.0) within the PAML package^73^. The estimated divergence times for *V. vinifera*-*H. annuus* (111–131 Mya), *S. lycopersicum*-*D. carota* (95–106 Mya) and *L. sativa*-*A. annua* (34–40 Mya) in TimeTree (http://www.timetree.org) were used to calibrate the tree. The expansion and contraction of the gene families clustered by OrthoMCL in the eight plants were determined with CAFÉ (v4.2)^74^.

### WGD analysis

All-versus-all protein sequence comparisons were performed using BLASTP (*E*-value: 1e−05) to identify homologous gene pairs. Syntenic blocks within and between species were determined using MCScanX^30^. The Perl script ‘add_ka_and_ks_to_collinearity.pl’. implemented in the MCScanX package was used to calculate the *Ks* values of the collinear homologous gene pairs. For five Asteraceae species, the neutral substitution rate of asterids (*r* = 8.25E−9) was applied to calculate the divergence date of the WGD or speciation events^24^. A Stevia-lettuce-sunflower syntenic block diagram and dot plot of coding genes between Stevia and sunflower were drawn with TBtools^75^. An image of the syntenic blocks and genomic features in the Stevia genome was produced with Circos (v0.69)^76^.

## Supplementary information

Supplementary Tables 1-11

Supplementary Figures 1-8Our article only contains two supplementary files, Supplementary Tables 1-11 and Supplementary Figures 1-8. Please delete the duplicate supplementary files, thanks.

## Data Availability

The Illumina short reads and PacBio long reads have been deposited in the NCBI SRA database under BioProject ID PRJNA684944. The transcriptome data have been deposited in the NCBI SRA database under BioProject ID PRJNA705537. The final chromosome-scale genome assembly and annotation data have been deposited in the Figshare database (10.6084/m9.figshare.14169491.v1).

## References

[1] Wu D (2018). Glucose-regulated phosphorylation of TET2 by AMPK reveals a pathway linking diabetes to cancer. Nature.

[2] Yang, T. et al. Hydrophobic recognition allows the glycosyltransferase UGT76G1 to catalyze its substrate in two orientationsCrystallizing sugar science. *Nat. Commun*. **10**, 3214 (2019). 10.1038/s41467-019-11154-4 PMC6642264 31324778

[3] Ceunen S, Geuns JM (2013). Steviol glycosides: chemical diversity, metabolism, and function. J. Nat. Prod..

[4] Yadav AK, Singh S, Dhyani D, Ahuja PS (2011). A review on the improvement of stevia [*Stevia rebaudiana* (Bertoni)]. Can. J. Plant Sci..

[5] Chatsudthipong V, Muanprasat C (2009). Stevioside and related compounds: therapeutic benefits beyond sweetness. Pharmacol. Ther..

[6] Philippaert K (2017). Steviol glycosides enhance pancreatic beta-cell function and taste sensation by potentiation of TRPM5 channel activity. Nat. Commun..

[7] Ceunen S (2013). Diterpene glycosides from *Stevia phlebophylla* A. Gray. Carbohydr. Res..

[8] Brandle JE, Starratt AN, Gijzen M (1998). *Stevia rebaudiana*: Its agricultural, biological, and chemical properties. Can. J. Plant Sci..

[9] Geuns JMC (2003). Stevioside. Phytochemistry.

[10] Lemus-Mondaca R, Vega-Gálvez A, Zura-Bravo L, Ah-Hen K (2012). *Stevia rebaudiana* Bertoni, source of a high-potency natural sweetener: a comprehensive review on the biochemical, nutritional and functional aspects. Food Chem..

[11] Wolwer-Rieck U, May B, Lankes C, Wust M (2014). Methylerythritol and mevalonate pathway contributions to biosynthesis of mono-, sesqui-, and diterpenes in glandular trichomes and leaves of *Stevia rebaudiana* Bertoni. J. Agric. Food Chem..

[12] Richman AS (1999). Diterpene synthesis in *Stevia rebaudiana*: recruitment and up-regulation of key enzymes from the gibberellin biosynthetic pathway. Plant J..

[13] Humphrey TV, Richman AS, Menassa R, Brandle JE (2006). Spatial organisation of four enzymes from *Stevia rebaudiana* that are involved in steviol glycoside synthesis. Plant Mol. Biol..

[14] Brandle JE, Telmer PG (2007). Steviol glycoside biosynthesis. Phytochemistry.

[15] Kim KK, Sawa Y, Shibata H (1996). Hydroxylation of *ent*-kaurenoic acid to steviol in *Stevia rebaudiana* Bertoni-purification and partial characterization of the enzyme. Arch. Biochem. Biophys..

[16] Zi JC, Mafu S, Peters RJ (2014). To gibberellins and beyond! Surveying the evolution of (di)terpenoid metabolism. Annu. Rev. Plant Biol..

[17] Yamaguchi S (2008). Gibberellin metabolism and its regulation. Annu. Rev. Plant Biol..

[18] Nagatoshi M (2012). UGT75L6 and UGT94E5 mediate sequential glucosylation of crocetin to crocin in *Gardenia jasminoides*. FEBS Lett..

[19] Sun Y (2018). Diterpenoid UDP-Glycosyltransferases from Chinese Sweet Tea and Ashitaba Complete the Biosynthesis of Rubusoside. Mol. Plant.

[20] Brandle JE, Richman A, Swanson AK, Chapman BP (2002). Leaf Ests from *Stevia rebaudiana*: a resource for gene discovery in diterpene synthesis. Plant Mol. Biol..

[21] Richman A (2004). Functional genomics uncovers three glucosyltransferases involved in the synthesis of the major sweet glucosides of *Stevia rebaudiana*. Plant J..

[22] Burton JN (2013). Chromosome-scale scaffolding of de novo genome assemblies based on chromatin interactions. Nat. Biotechnol..

[23] Simão FA (2015). BUSCO: assessing genome assembly and annotation completeness with single-copy orthologs. Bioinformatics.

[24] Badouin H (2017). The sunflower genome provides insights into oil metabolism, flowering and Asterid evolution. Nature.

[25] Reyes-Chin-Wo S (2017). Genome assembly with in vitro proximity ligation data and whole-genome triplication in lettuce. Nat. Commun..

[26] Song C (2018). The *Chrysanthemum nankingense* Genome Provides Insights into the Evolution and Diversification of Chrysanthemum Flowers and Medicinal Traits. Mol. Plant.

[27] Soltis PS, Marchant DB, Van de Peer Y, Soltis DE (2015). Polyploidy and genome evolution in plants. Curr. Opin. Genet. Dev..

[28] Soltis PS, Soltis DE (2016). Ancient WGD events as drivers of key innovations in angiosperms. Curr. Opin. Plant. Biol..

[29] Soltis DE (2009). Polyploidy and angiosperm diversification. Am. J. Bot..

[30] Wang Y (2012). MCScanX: a toolkit for detection and evolutionary analysis of gene synteny and collinearity. Nucleic Acids Res..

[31] Barker MS (2016). Most Compositae (Asteraceae) are descendants of a paleohexaploid and all share a paleotetraploid ancestor with the Calyceraceae. Am. J. Bot..

[32] Jiao Y (2012). A genome triplication associated with early diversification of the core eudicots. Genome Biol..

[33] Kumar H (2012). A comprehensive analysis of fifteen genes of steviol glycosides biosynthesis pathway in *Stevia rebaudiana* (Bertoni). Gene.

[34] Sapir-Mir M (2008). Peroxisomal localization of *Arabidopsis* isopentenyl diphosphate isomerases suggests that part of the plant isoprenoid mevalonic acid pathway is compartmentalized to peroxisomes. Plant Physiol..

[35] Wang J, Li S, Xiong Z, Wang Y (2016). Pathway mining-based integration of critical enzyme parts for de novo biosynthesis of steviolglycosides sweetener in Escherichia coli. Cell Res..

[36] Nomura T (2013). Functional analysis of *Arabidopsis* CYP714A1 and CYP714A2 reveals that they are distinct gibberellin modification enzymes. Plant Cell Physiol..

[37] Tandel KR (2011). Sugar substitutes: health controversy over perceived benefits. J. Pharmacol. Pharmacother..

[38] Swithers SE (2013). Artificial sweeteners produce the counterintuitive effect of inducing metabolic derangements. Trends Endocrinol. Metab..

[39] O’Neill K, Pirro S (2020). The complete genome sequence of *Stevia rebaudiana*, the Sweetleaf. F1000Research..

[40] Renny-Byfield S, Wendel JF (2014). Doubling down on genomes: polyploidy and crop plants. Am. J. Bot..

[41] Panchy N, Lehti-Shiu M, Shiu SH (2016). Evolution of gene duplication in plants. Plant Physiol..

[42] Koren S (2017). Canu: scalable and accurate long-read assembly via adaptive k-mer weighting and repeat separation. Genome Res..

[43] Chin CS (2016). Phased diploid genome assembly with single-molecule real-time sequencing. Nat. Methods.

[44] Chakraborty M, Baldwin-Brown JG, Long AD, Emerson JJ (2016). Contiguous and accuratede novoassembly of metazoan genomes with modest long read coverage. Nucleic Acids Res..

[45] Walker BJ (2014). Pilon: an integrated tool for comprehensive microbial variant detection and genome assembly improvement. PLoS ONE.

[46] Li H, Durbin R (2009). Fast and accurate short read alignment with Burrows-Wheeler transform. Bioinformatics.

[47] Parra G, Bradnam K, Korf I (2007). CEGMA: a pipeline to accurately annotate core genes in eukaryotic genomes. Bioinformatics.

[48] Belton JM (2012). Hi-C: A comprehensive technique to capture the conformation of genomes. Methods.

[49] Servant N (2015). HiC-Pro: an optimized and flexible pipeline for Hi-C data processing. Genome Biol..

[50] Edgar RC, Myers EW (2005). PILER: identification and classification of genomic repeats. Bioinformatics.

[51] Price AL, Jones NC, Pevzner PA (2005). De novo identification of repeat families in large genomes. Bioinformatics.

[52] Xu Z, Wang H (2007). LTR_FINDER: an efficient tool for the prediction of full-length LTR retrotransposons. Nucleic Acids Res..

[53] Wicker T (2007). A unified classification system for eukaryotic transposable elements. Nat. Rev. Genet..

[54] Tarailo-Graovac M, Chen N (2009). Using RepeatMasker to identify repetitive elements in genomic sequences. Curr. Protoc. Bioinforma..

[55] Jurka J (2005). Repbase Update, a database of eukaryotic repetitive elements. Cytogenet Genome Res..

[56] Stanke M, Steinkamp R, Waack S, Morgenstern B (2004). AUGUSTUS: a web server for gene finding in eukaryotes. Nucleic Acids Res..

[57] Majoros WH, Pertea M, Salzberg SL (2004). TigrScan and GlimmerHMM: two open source ab initio eukaryotic gene-finders. Bioinformatics.

[58] Bromberg Y, Rost B (2007). SNAP: predict effect of non-synonymous polymorphisms on function. Nucleic Acids Res..

[59] Blanco E, Parra G, Guigó R (2007). Using geneid to identify genes. Curr. Protoc. Bioinforma..

[60] Altschul SF (1990). Basic local alignment search tool. J. Mol. Biol..

[61] Keilwagen J (2016). Using intron position conservation for homology-based gene prediction. Nucleic Acids Res..

[62] Tang S, Lomsadze A, Borodovsky M (2015). Identification of protein coding regions in RNA transcripts. Nucleic Acids Res..

[63] Haas BJ (2008). Automated eukaryotic gene structure annotation using EVidenceModeler and the Program to Assemble Spliced Alignments. Genome Biol..

[64] Campbell MA (2006). Comprehensive analysis of alternative splicing in rice and comparative analyses with Arabidopsis. BMC Genomics.

[65] Boeckmann B (2003). The SWISS-PROT protein knowledgebase and its supplement TrEMBL in 2003. Nucleic Acids Res..

[66] Conesa A (2005). Blast2GO: a universal tool for annotation, visualization and analysis in functional genomics research. Bioinformatics.

[67] Ashburner M (2000). Gene ontology: tool for the unification of biology. The Gene Ontology Consortium. Nat. Genet..

[68] Kanehisa M, Goto S (2000). KEGG: kyoto encyclopedia of genes and genomes. Nucleic Acids Res..

[69] Li L, Stoeckert CJ, Roos DS (2003). OrthoMCL: identification of ortholog groups for eukaryotic genomes. Genome Res..

[70] Katoh K, Standley DM (2013). MAFFT multiple sequence alignment software version 7: improvements in performance and usability. Mol. Biol. Evol..

[71] Castresana J (2000). Selection of conserved blocks from multiple alignments for their use in phylogenetic analysis. Mol. Biol. Evol..

[72] Stamatakis A (2006). RAxML-VI-HPC: maximum likelihood-based phylogenetic analyses with thousands of taxa and mixed models. Bioinformatics.

[73] Yang Z (2007). PAML 4: phylogenetic analysis by maximum likelihood. Mol. Biol. Evol..

[74] De Bie T (2006). CAFE: a computational tool for the study of gene family evolution. Bioinformatics.

[75] Chen C (2020). TBtools: An Integrative Toolkit Developed for Interactive Analyses of Big Biological Data. Mol. Plant.

[76] Krzywinski M (2009). Circos: An information aesthetic for comparative genomics. Genome Res..

