# Supplementary material for: The chromosome-level Stevia genome provides insights into steviol glycoside biosynthesis
Source: Hortic Res. 2021 Jun 1;8:129. doi: 10.1038/s41438-021-00565-4 (PMC8166950; doi:10.1038/s41438-021-00565-4)
Supplement: Supplementary file 1 — Supplementary Tables 1-11 [file 41438_2021_565_MOESM1_ESM.docx]

**Supplementary Tables 1-11**

**Supplementary Table 1. K-mer analysis of the Steiva genome by using K-mer = 19.**

| **K-mer** | **K-mer number** | **K-mer Depth (X)** | **Genome Size (Mb)** | **Heterozygous Ratio (%)** | **Repeat (%)** |
| --- | --- | --- | --- | --- | --- |
| 19 | 51,963,761,089 | 45 | 1,155 | 0.43 | 73.13 |

**Supplementary Table 2. Statistics of PacBio sequencing data.**

| **Subreads number** | **Total data (bp)** | **Mean reads length (bp)** | **N50 (bp)** | **Coverage (X)** |
| --- | --- | --- | --- | --- |
| 13,862,894 | 114,957,745,511 | 8,292 | 12,823 | 99.53 |

**Supplementary Table 3. Statistics of pre-assembly of the Steiva genome.**

| **Contig number** | **Contig length (bp)** | **Contig N50 (bp)** | **Contig max (bp)** | **GC content (%)** |
| --- | --- | --- | --- | --- |
| 6,978 | 1,404,649,072 | 616,850 | 26,267,939 | 36.97 |

**Supplementary Table 4. Statistics of Hi-C data and assessment.**

| **Statistics of Hi-C data** | | | |
| --- | --- | --- | --- |
| Number of read pairs | Number of bases (bp) | GC content (%) | % ≥ Q30 |
| 256,637,612 | 76,864,667,140 | 37.86 | 90.92 |
| **Statistics of mapping** | | | |
| Mapping type | | Number of reads | Ratio (%) |
| Total read pairs | | 256,637,612 | 100 |
| Mapped reads | | 464,105,604 | 90.42 |
| Unique mapped read pairs | | 63,522,997 | 24.75 |
| **Statistics of valid Hi-C data** | | | |
| Type | | Number of reads | Ratio (%) |
| Unique paired alignments | | 63,522,997 | 100 |
| Valid interaction pairs | | 47,406,087 | 74.63 |
| Dangling end pairs | | 8,834,230 | 13.91 |
| Re-ligation pairs | | 1,208,602 | 1.9 |
| Self-cycle pairs | | 1,444,844 | 2.27 |
| Dumped pairs | | 4,629,234 | 7.29 |

**Supplementary Table 5. Summary of chromosome level assembly based on Hi-C data.**

| **Chromosome** | **Number of anchored contigs** | **Length of clustered contigs (bp)** |
| --- | --- | --- |
| Chr1 | 866 | 177,034,398 |
| Chr2 | 962 | 191,578,415 |
| Chr3 | 394 | 85,830,139 |
| Chr4 | 644 | 123,644,549 |
| Chr5 | 502 | 121,893,487 |
| Chr6 | 220 | 104,072,746 |
| Chr7 | 494 | 114,979,049 |
| Chr8 | 525 | 113,396,820 |
| Chr9 | 584 | 112,665,887 |
| Chr10 | 556 | 107,100,625 |
| Chr11 | 611 | 127,849,971 |
| Total Sequences Clustered (%) | 6,358 (83.90%) | 1,380,046,086 (98.26%) |
| Total Sequences Ordered and Oriented (%) | 3,864(60.77%) | 1,259,684,888 (91.28%) |

**Supplementary Table 6.** **Statistics of** **Illumina clean reads mapping rate of the Steiva genome assembly.**

| **Library** | **Total reads** | **Mapped reads** | **Mapped (%)** | **Properly mapped reads** | **Properly_mapped (%)** |
| --- | --- | --- | --- | --- | --- |
| 270_1 bp | 212,630,443 | 207,696,587 | 97.68% | 197,178,446 | 93.71% |
| 270_2 bp | 211,582,947 | 208,639,238 | 98.61% | 197,812,570 | 94.50% |

**Supplementary Table 7. Quality assessment of the assembled genome of the Steiva using CEMGA.**

| **Species** | **Number of 458 CEGs* present in assembly** | **% of 458 CEGs present in assemblies** | **Number of 248 highly conserved CEGs present** | **% of 248 highly conserved CEGs present** |
| --- | --- | --- | --- | --- |
| ***S. rebaudiana*** | 451 | 98.47% | 242 | 97.58% |

**Supplementary Table 8. Quality assessment of the assembled genome of the Steiva using BUSCOs.**

| **Type** | **Number** | **Percent (%)** |
| --- | --- | --- |
| Complete BUSCOs | 1,239 | 86.04 |
| Complete and single-copy BUSCOs | 983 | 68.26 |
| Complete and duplicated BUSCOs | 256 | 17.78 |
| Fragmented BUSCOs | 40 | 2.78 |
| Missing BUSCOs | 161 | 11.18 |
| Total BUSCO groups searched | 1,440 | 100 |

**Supplementary Table 9. Classification of repetitive elements in the Steiva genome.**

| **Repeat type** | **Number of elements** | **Length（bp）** | **Percentage in Genome (%)** |
| --- | --- | --- | --- |
| Class I: Retrotransposons | **1,103,869** | **98,352,7546** | **69.45** |
| LTR-Retrotransposons | 653,092 | 921,487,649 | 65.07 |
| LTR/Copia | 229,238 | 297,603,234 | 21.02 |
| LTR/Gypsy | 41,3942 | 615,228,300 | 43.44 |
| LTR-other | 9,912 | 8,656,115 | 0.61 |
| Non-LTR Retrotransposons | 38,020 | 14,351,593 | 1.01 |
| LINE | 32,671 | 13,360,099 | 0.94 |
| SINE | 5,349 | 991,494 | 0.07 |
| Other Retrotransposons | 412,757 | 47,688,304 | 3.37 |
| Class II: DNA Transposons | **205,834** | **82,493,967** | **5.83** |
| Crypton | 5 | 316 | 0.00 |
| Helitron | 34,874 | 12,532,583 | 0.88 |
| MITE | 6,778 | 1,600,808 | 0.11 |
| Maverick | 3,653 | 1,105,403 | 0.08 |
| TIR | 155,633 | 65,826,590 | 4.65 |
| Other | 4,891 | 1,428,267 | 0.10 |
| Potential Host Genes | **31,645** | **7,812,408** | **0.55** |
| SSR | **3,612** | **1,152,290** | **0.08** |
| Unknown | **206,408** | **59,495,291** | **4.20** |
| Total | 1,551,368 | 113,4481,502 | 80.11 |

**Supplementary Table 10. Functional annotation of predicted protein-coding genes in the Steiva genome.**

| **Database** | **Numbers of matching genes** | **Percent of annotated genes (%)** |
| --- | --- | --- |
| GO | 22,116 | 50.10% |
| KEGG | 13,589 | 30.78% |
| KOG | 24,024 | 54.42% |
| TrEMBL | 41,719 | 94.51% |
| NR | 41,754 | 94.59% |
| Total | 41,800 | 94.69% |

**Supplementary Table 11. Summary of gene family clustering.**

| **Species** | **Total genes** | **Families** | **Genes in family** | **Unique families** | **Genes in unique families** |
| --- | --- | --- | --- | --- | --- |
| *V. vinifera* | 26,346 | 14,209 | 18,796 | 750 | 2,380 |
| *S. lycopersicum* | 34,725 | 16,411 | 24,828 | 2,458 | 7,747 |
| *D. carota* | 32,115 | 15,945 | 25,765 | 1,325 | 4,614 |
| *L. sativa* | 38,910 | 20,047 | 31,630 | 1,237 | 4,797 |
| *C. nankingense* | 56,870 | 22,034 | 45,408 | 1,821 | 7,607 |
| *A. annua* | 63,226 | 23,509 | 49,218 | 2,320 | 9,662 |
| *H. annuus* | 52,243 | 21,804 | 40,418 | 1,583 | 5,002 |
| *S. rebaudiana* | 44,143 | 20,147 | 40,214 | 1,057 | 4,281 |
