# Supplementary material for: The chromosome-level Stevia genome provides insights into steviol glycoside biosynthesis
Source: Hortic Res. 2021 Jun 1;8:129. doi: 10.1038/s41438-021-00565-4 (PMC8166950; doi:10.1038/s41438-021-00565-4)
Supplement: Supplementary file 2 — Supplementary Figures 1-8Our article only contains two supplementary files, Supplementary Tables 1-11 and Supplementary Figures 1-8. Please delete the duplicate supplementary files, thanks. [file 41438_2021_565_MOESM2_ESM.docx]

**Supplementary Figures 1-8**


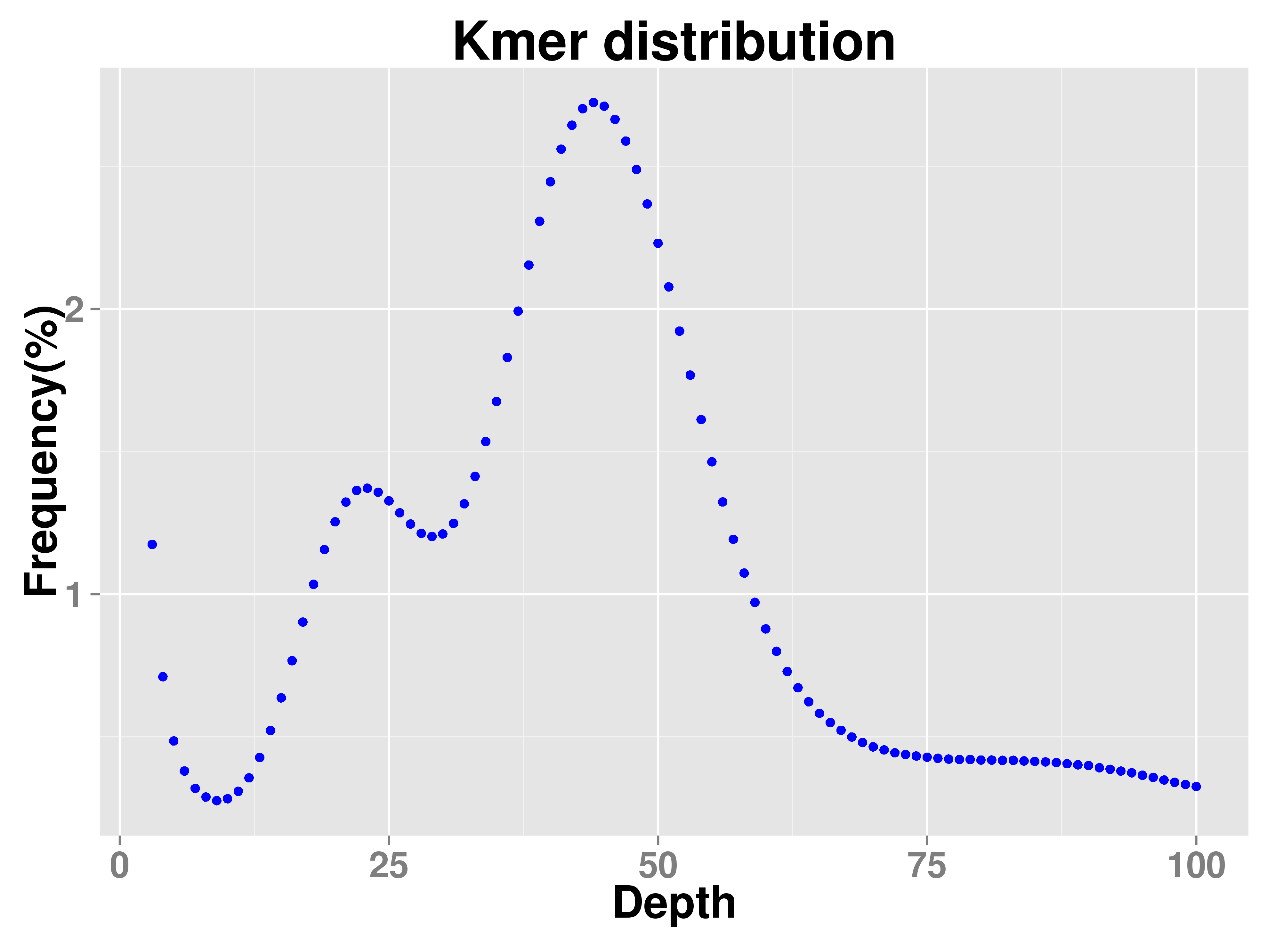


**Supplementary Figure 1. Estimation of the Stevia genome size by K-mer analysis.** The figure shows the frequency of 19 k-mers, which are 19 bp sequences from clean reads of short-insert-size libraries. We identified 51,963,761,089 K-mers and the peak of K-mer depth is 45. Genome size can be estimated as (total K-mer number) / (the volume peak). The genome size of the Stevia was thus estimated as 1,155 Mb.


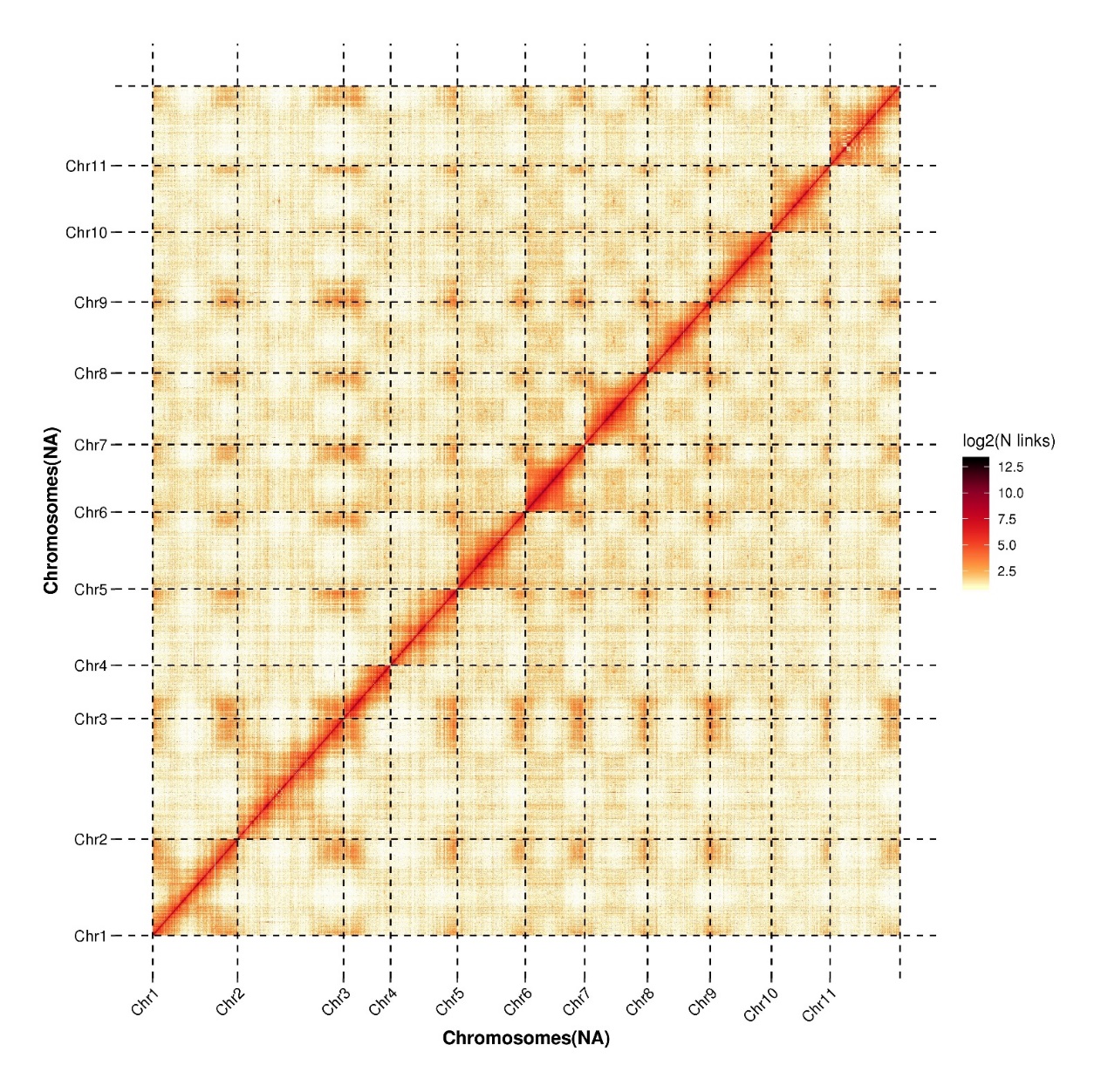


**Supplementary Figure** **2.** **Hi-C map of the Stevia genome showing genome-wide all-by-all interactions under a resolution of 200 kb.**


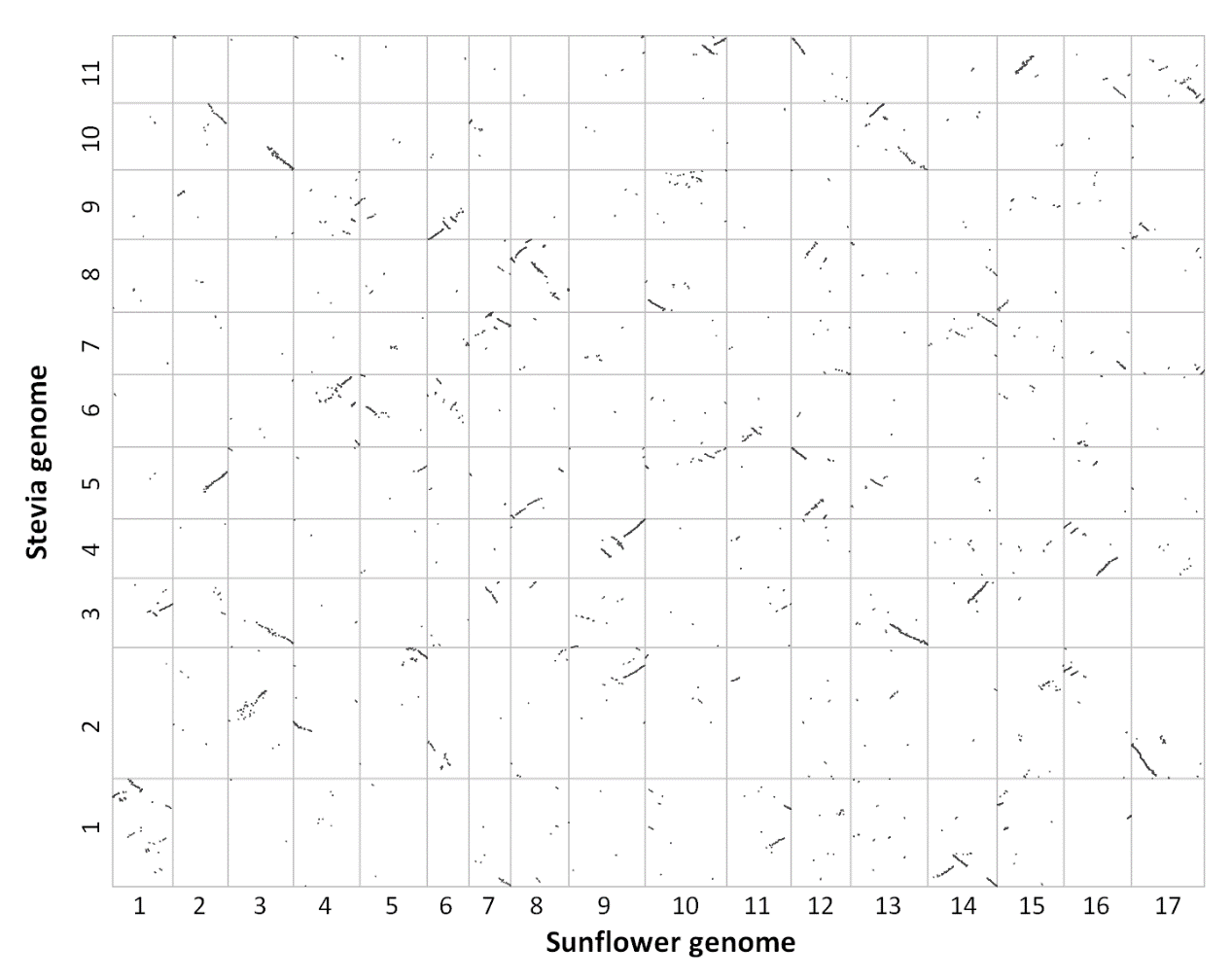


**Supplementary Figure** **3.** **Dot plot illustrating the comparative analysis between the Stevia and sunflower genomes.**


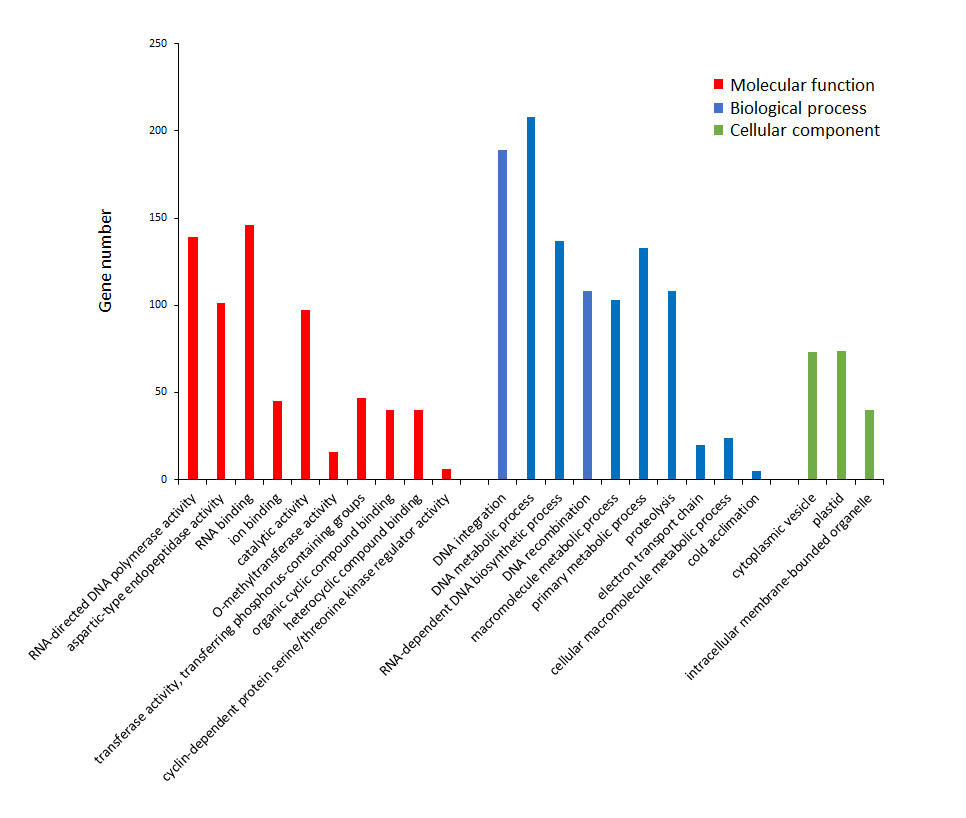


**Supplementary Figure 4.** **Enriched GO terms for gene families specific to Stevia.**


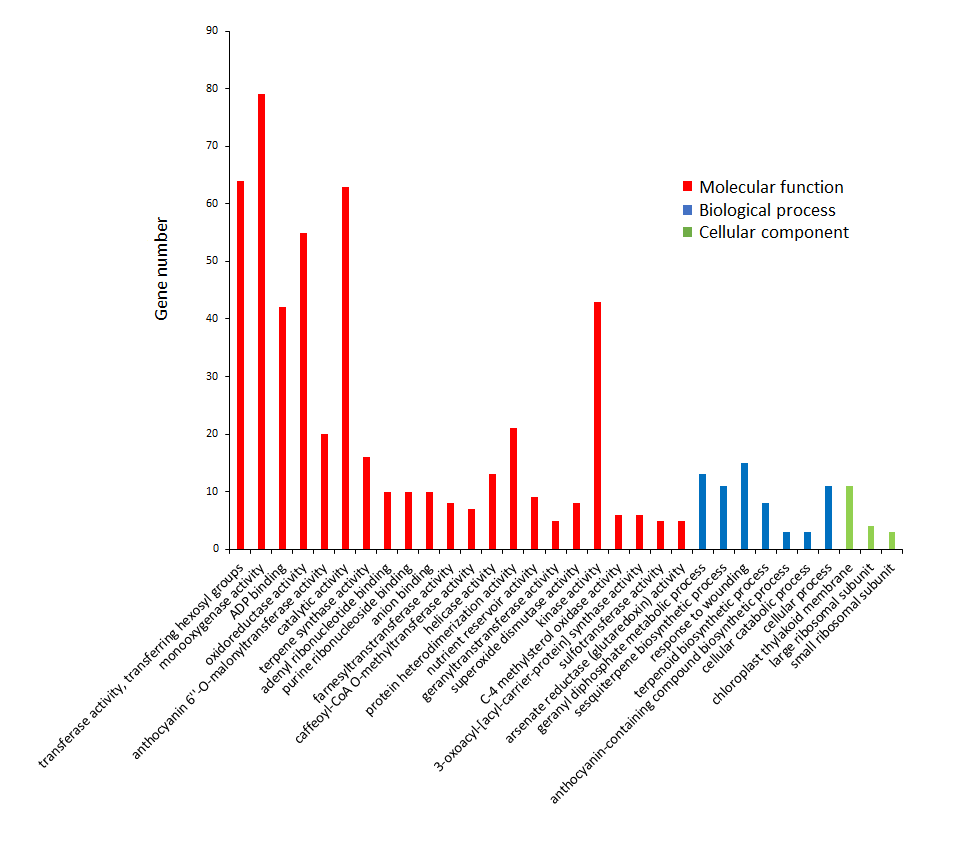


**Supplementary Figure 5. Enriched GO terms for gene families expanded in Stevia.**


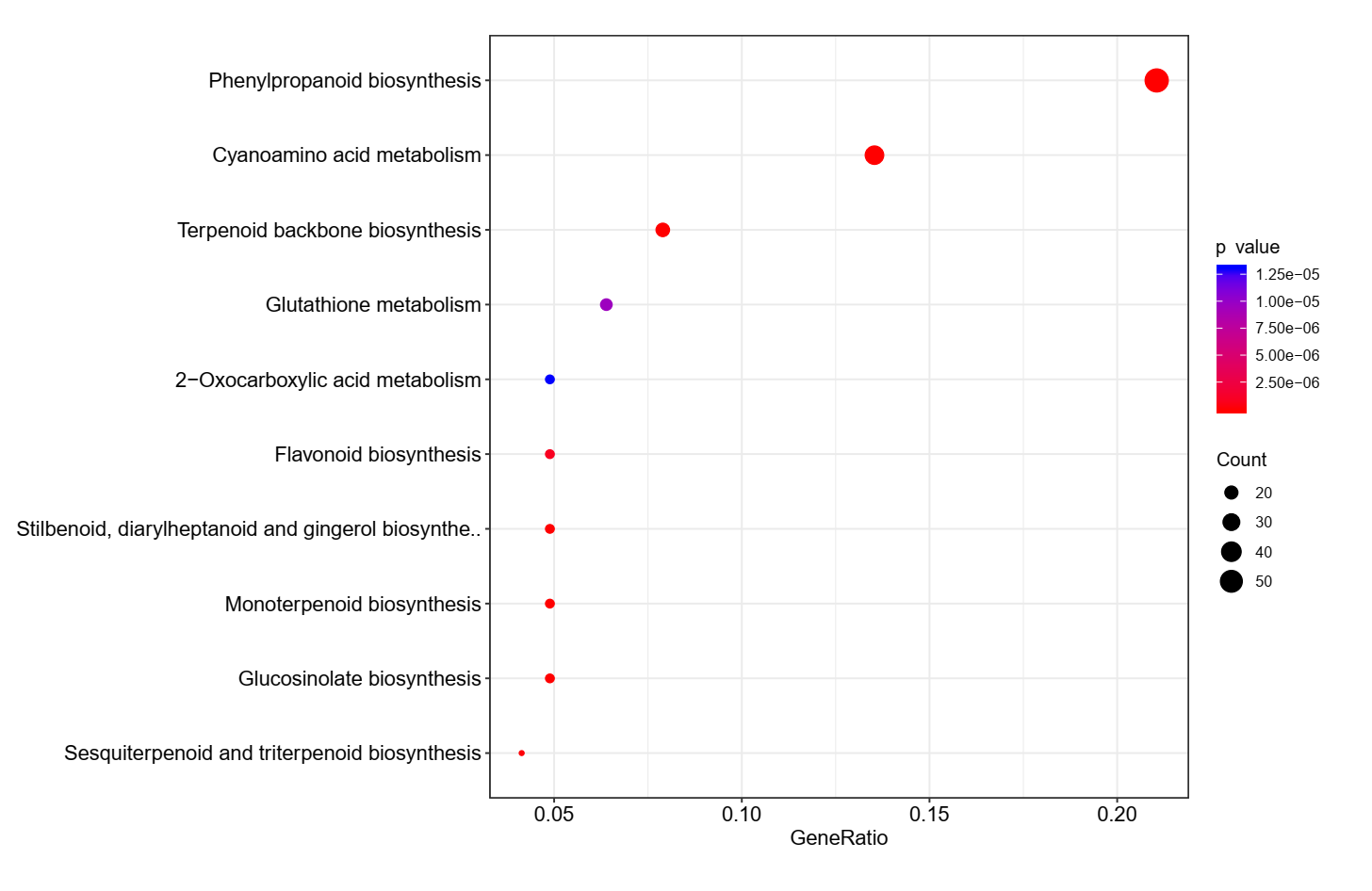


**Supplementary Figure 6. Enriched KEGG** **pathways for gene families expanded in Stevia.**


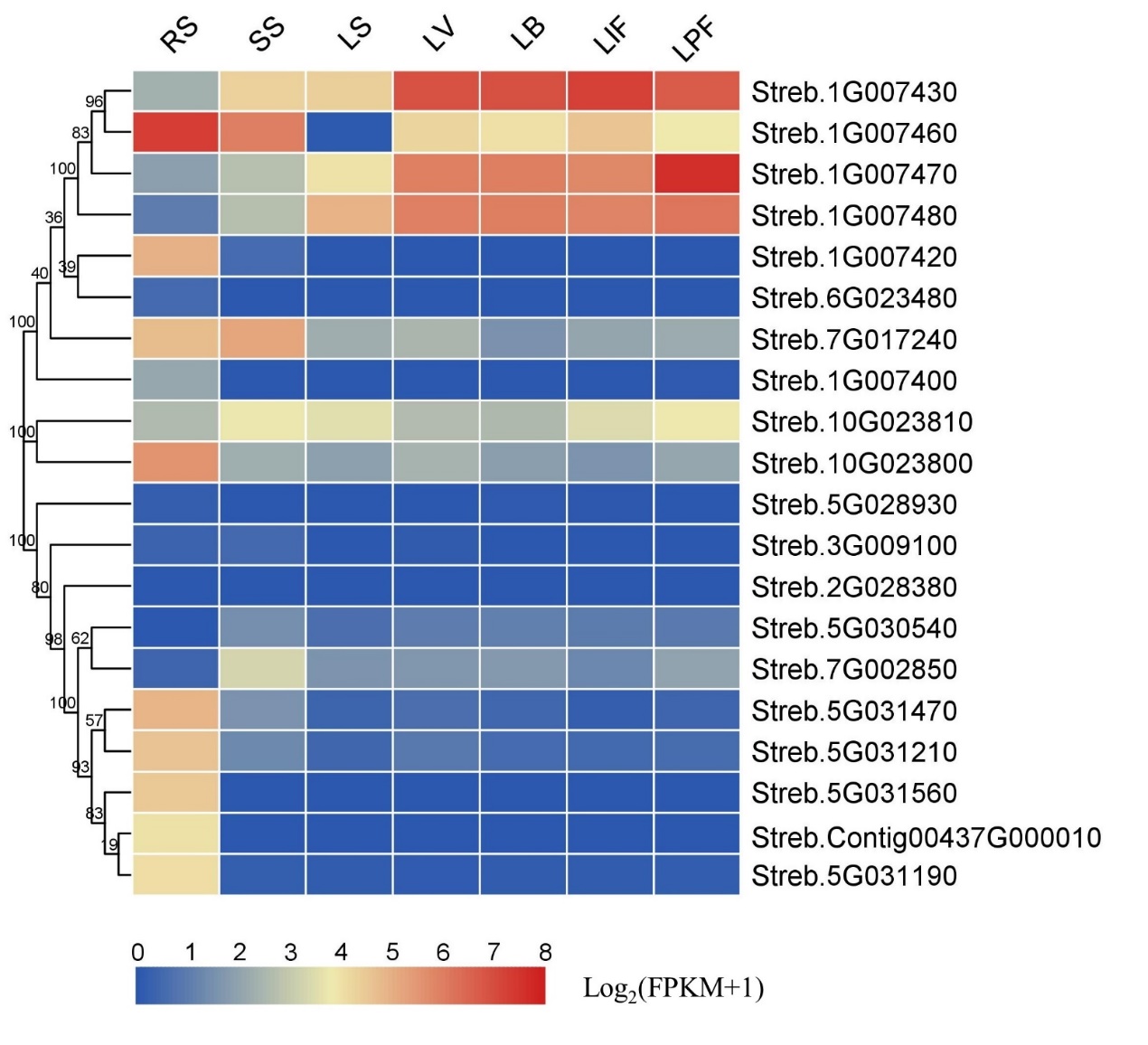
**Supplementary Figure 7. Expression profiles of CYP716 family genes in Stevia.** RS: root at the seedling stage; SS: stem at the seedling stage; LS: leaf at the seedling stage; LV: leaf at the vegetative stage; LB: leaf at bud stage; LIF: leaf at the initial flowering stage; LPF: leaf at the peak flowering stage.


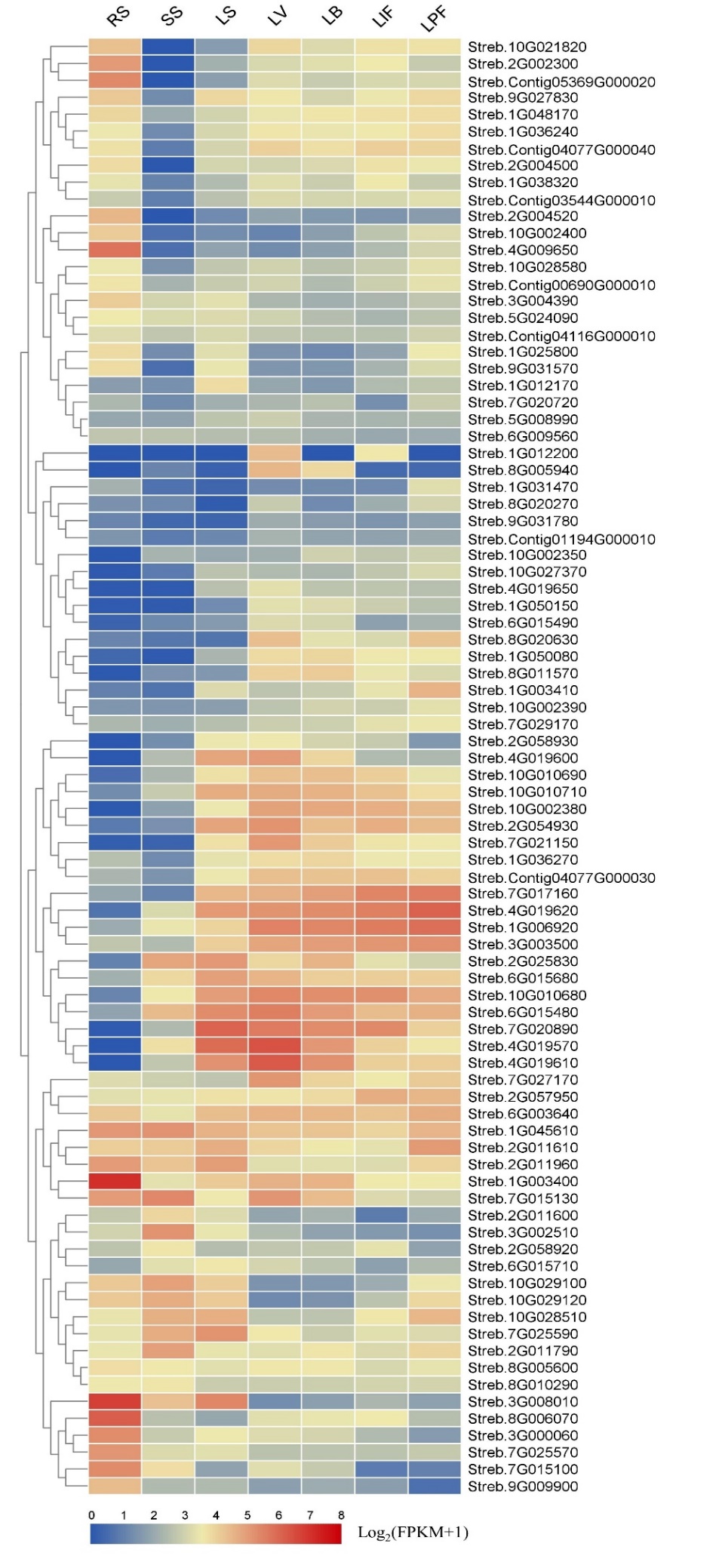


**Supplementary Figure 8. Expression profiles of selected UGT genes in Stevia.** RS: root at the seedling stage; SS: stem at the seedling stage; LS: leaf at the seedling stage; LV: leaf at the vegetative stage; LB: leaf at bud stage; LIF: leaf at the initial flowering stage; LPF: leaf at the peak flowering stage.
